# BAFF/APRIL System Is Functional in B-Cell Acute Lymphoblastic Leukemia in a Disease Subtype Manner

**DOI:** 10.3389/fonc.2019.00594

**Published:** 2019-07-18

**Authors:** Eirini Sevdali, Eleni Katsantoni, Cristian R. Smulski, Maria Moschovi, Maria Palassopoulou, Eleni-Nefeli Kolokotsa, Nikoletta Argentou, Nikolaos Giannakoulas, Maria Adamaki, Georgios Vassilopoulos, Sophia Polychronopoulou, Anastasios E. Germenis, Hermann Eibel, Matthaios Speletas

**Affiliations:** ^1^Department of Immunology and Histocompatibility, Faculty of Medicine, School of Health Sciences, University of Thessaly, Larissa, Greece; ^2^Basic Research Center, Biomedical Research Foundation of the Academy of Athens, Athens, Greece; ^3^Center for Chronic Immunodeficiency, University Medical Center Freiburg, Freiburg im Breisgau, Germany; ^4^Hematology/Oncology Unit, First Department of Pediatrics, National and Kapodistrian University of Athens, Medical School, “Aghia Sophia” Children's Hospital, Athens, Greece; ^5^Department of Hematology, Faculty of Medicine, School of Health Sciences, University of Thessaly, Larissa, Greece; ^6^Department of Pediatric Hematology/Oncology, “Aghia Sophia” Children's Hospital, Athens, Greece

**Keywords:** BAFFR, B-ALL, E2A-PBX1, glucocorticoids, apoptosis

## Abstract

BAFF, APRIL and their receptors regulate the survival, maturation and homeostasis of mature B-cells. Despite the lack of a functional role of BAFF/APRIL system during normal early B-cell development, previous studies indicated a contribution of these molecules in the pathogenesis of B-lineage acute lymphoblastic leukemia (B-ALL). Here, we evaluated the expression of this system in B-ALL and its involvement in spontaneous and drug-induced apoptosis of B-lymphoblasts, taking into consideration the distinct disease subtypes. We found that BAFFR is the most predominant aberrantly expressed receptor in B-ALL and that its expression, along with BCMA and APRIL, positively correlates with the maturation stage of B-lymphoblasts. Moreover, the binding of the E2A-PBX1 chimeric protein to the *BAFFR* promoter suggests that the transcriptional activator promotes the increase in *BAFFR* expression observed in about 50% of pre-B-ALL patients carrying the *t*_(1, 19)_ translocation. BAFF binding to BAFFR led to the processing of NF-κB2 p100 in pre-B ALL cells suggesting that BAFFR can activate the NF-κB2 pathway in pre-B ALL cells. Surprisingly, we found that BAFF treatment promotes the cell death of primary BCR-ABL^+^ BAFFR^+^ pre-B-lymphoblasts in adult B-ALL. It also enhances glucocorticoid-induced apoptosis in the E2A-PBX1^+^ pre-B-ALL cell line 697. These data suggest that BAFF/BAFFR signaling in B-ALL cells differs from normal B cells and that it may affect the pathogenesis of the disease.

## Introduction

B-cell activating factor (BAFF), a member of the tumor necrosis factor (TNF) family, is an essential survival factor for B-cells. BAFF is produced as a membrane-bound protein and upon proteolytic cleavage it is released as a soluble ligand, which exists either as trimer or 60-mer, which combines 20 BAFF trimers to form a virus-like structure (1–4). BAFF binds to three receptors differentially expressed by mature B-cell subsets (5). BAFF receptor (BAFFR), which only binds BAFF, is expressed at high levels by all B-cell subsets from the stage of transitional B-cell except for germinal center B-cells and plasma cells. The transmembrane activator, calcium modulator and cyclophilin ligand interactor (TACI) is expressed by marginal zone B-cells, switched memory B-cells and plasma cells, while the B-cell maturation antigen (BCMA) is expressed by plasma cells. TACI and BCMA can bind BAFF and APRIL (“a proliferation-inducing ligand”), although at different affinities (6). Since BAFFR does not seem to be expressed before the stage of IgM^+^ immature B-cells (7, 8) and because inactivation of BAFF and BAFFR still allows normal development of B-cell precursors up to the stage of transitional B-cells (9, 10), BAFF and BAFFR are not required for the normal development of pro- and pre-B cells during these early stages of B-cell development.

Several reports provided evidence that the BAFF/APRIL system also plays a role in the pathogenesis of mature B-lineage cancer (11–13) as the receptors and the ligands are expressed by neoplastic B-cells and as the tumor microenvironment can provide BAFF and APRIL to support the survival of tumor B-cells (14). Moreover, it has been suggested that the BAFF/APRIL system also contributes to the pathogenesis of B-cell acute lymphoblastic leukemia (B-ALL) (15–19), the most common malignancy of childhood. Interestingly, these studies focused on the aberrant expression of BAFFR, since this receptor seems to be mainly expressed by malignant B-lymphoblasts (17–19). In this context, Fazio et al. observed a positive selection and survival of BAFFR-expressing blasts (16), while high levels of ligands were reported to provide survival signals, protecting malignant B-lymphoblasts from drug-induced apoptosis (18, 19). These findings have led several groups to investigate novel therapeutic approaches by targeting BAFFR either with toxin-fused antibodies (20, 21) or, more recently, through a CAR-based approach (22).

Since B-ALL is a heterogeneous type of leukemia, different mechanisms may account for the aberrant expression and function of BAFF/APRIL system molecules. For example, the chromosomal translocation t(1;19) is found in ~50% of pre-B ALL cases and correlates with intermediate prognosis. The translocation leads to the expression of a protein which combines the transactivation domains of the transcription factor E2A with the DNA binding domain of PBX1 (23). Here we show that the chimeric E2A-PBX1 protein binds to the promoter of *BAFFR* gene in pre-B-lymphoblasts, suggesting that E2A-PBX1 plays a role in the upregulation of BAFFR expression. Moreover, the aberrantly expressed BAFFR surprisingly acts as a cell death-promoting receptor of pre-B-ALL cells.

## Materials and Methods

### Subjects and Cell Lines

Seventy-one patients with ALL (59 children, 12 adults), diagnosed according to standard criteria (24, 25), were enrolled in the study. Their demographic and clinicolaboratory characteristics are presented in Table 1. Bone marrow (BM) aspirates performed in 9 individuals for diagnostic purposes (including 4 patients with high grade non-Hodgkin lymphoma during follow-up and far off any therapy, at least 1 year after a complete remission was achieved), as well as peripheral blood (PB) samples from 11 healthy subjects served as controls.

**Table 1:** Demographic data and clinical characteristics of the patients of the study.

	**Total ALL**	**T-ALL**	**Total B-ALL**	**Pediatric B-ALL**	**Adult B-ALL**	**Healthy donors**
**No**	71	7	64	52	12	20
**Sex, male/female**	41/30	4/3	37/27	32/20	5/7	5/15
**Age, years**	6.0	7.0	6.0	4.4	50.0	44.5
**Median (Range)**	(0.8–77.0)	(3.0–13.5)	(0.8–77.0)	(0.8–14.0)	(20.0–77.0)	(0.5–72)
**All Subtypes**						
**B phenotype**, ***n*** **(%)**	64 (90.1)					
pro–B, *n* (%)	2 (2.8)		2 (3.1)	1 (1.9)	1 (8.3)	
Common B, *n* (%)	39 (54.9)		39 (60.9)	35 (67.3)	4 (33.3)	
Pre–B, *n* (%)	23 (32.4)		23 (35.9)	16 (30.8)	7 (58.3)	
**T Phenotype**, ***n*** **(%)**	7 (9.9)					
cortical T, *n* (%)	3 (4.2)	3 (42.9)				
pre–T, *n* (%)	2 (2.8)	2 (28.6)				
mature T, *n* (%)	2 (2.8)	2 (28.6)				
**WBC count, x10**^**9**^**/L**	14.1	164.1	11.9	10.3	20.5	
**Median (range)**	(0.3–547.3)	(10.1–547.3)	(0.3–108.3)	(0.3–108.3)	(2.0–80.0)	
**Hemoglobin, mg/dL**	9.0	9.8	8.8	8.4	10.8	
**Median (range)**	(3.0–14.5)	(8.1–12.7)	(3.0–14.5)	(3.0–13.4)	(4.3–14.5)	
**Platelets, x10**^**9**^**/L**	80.0	85.0	79.0	75.0	92.0	
**Median (range)**	(9.0–952.0)	(20.0–316.0)	(9.0–952.0)	(9.0–952.0)	(10.0–380.0)	
**Bone marrow infiltration,%**	73.5	77.0	72.7	71.0	86.9	
**Median (range)**	(16.0–98.0)	(68.0–94.0)	(16.0–98.0)	(16.0–98.0)	(50.0–95.0)	
**Immunophenotyping**						
aberrant CD13/33, *n* (%)		0 (0.0)	8 (12.5)	5 (9.6)	3 (25.0)	
aberrant T markers, *n* (%)			0 (0.0)	0 (0.0)	0 (0.0)	
aberrant B markers, *n* (%)		1 (14.3)				
**Karyotype**^†^						
Hyperdiploidy, *n* (%)	2 (4.3)	0 (0.0)	2 (4.7)	2 (6.5)	0 (0.0)	
Highly hyperdipl., *n* (%)	8 (17.0)	0 (0.0)	8 (18.6)	8 (25.8)	0 (0.0)	
Hypodiploidy, *n* (%)	1 (2.1)	0 (0.0)	1 (2.3)	1 (3.2)	0 (0.0)	
Normal karyotype, *n* (%)	18 (38.3)	0 (0.0)	18 (41.9)	12 (38.7)	5 (41.7)	
Other defects, *n* (%)	21 (44.7)	4 (100.0)	17 (16.3)	11 (35.5)	7 (58.3)	
**Molecular Defects ^†^**						
E2A–PBX1, *n* (%)	7 (10)	0 (0.0)	7 (11.1)	5 (9.6)	2 (18.2)	
TEL–AML1, *n* (%)	18 (25.7)	0 (0.0)	18 (28.6)	18 (34.6)	0 (0.0)	
BCR–ABL, *n* (%)	5 (7.1)	0 (0.0)	5 (7.9)	2 (3.8)	3 (27.3)	
**Early Response**, ***n*** **(%)**^†^						
Complete remission, *n* (%)	64 (92.8)	7 (100)	57 (91.9)	50 (98.0)	7 (63.6)	
Partial remission, *n* (%)	2 (2.9)	0 (0.0)	2 (3.2)	1 (2.0)	1 (9.1)	
Resistant disease, *n* (%)	3 (4.3)	0 (0.0)	3 (4.8)	0 (0.0)	3 (27.3)	
**Relapse, n (%)**^†^	7 (10.6)	0 (0.0)	7 (11.9)	5 (9.8)	2 (25.0)	
**Death**, ***n*** **(%)**	12 (17.1)	1 (14.3)	11 (17.5)	5 (9.8)	6 (50.0)	
**Survival, in months**	89.0	96.0	87.0	93.0	10.0	
**Median (range)**	(1.0–201.0)	(2.0–193.0)	(1.0–201.0)	(3.0–201.0)	(1.0–45.0)	

† *Non available data: 24 cases for karyotype, 1 case for molecular defects, 2 cases for early response and relapse*.

Mononuclear cells were obtained by density-gradient centrifugation from 70 out of 71 patients with ALL, from 4 BM samples of patients with non-Hodgkin lymphoma of the control group and from all (11) normal PB samples. For B-cell survival assays, B-lymphoblasts, and normal PB B-cells were isolated following magnetic depletion of all non-CD19^+^ subsets. For the mRNA analysis of BAFF/APRIL ligands and receptors, monocytes, and B-cells were purified from normal PB mononuclear cells. Details on cell isolation are provided in Supplementary Methods.

The pre-B-ALL cell line 697 (E2A-PBX1^+^) and the T-ALL cell line Jurkat were cultured in IMDM, supplemented with 10% heat-inactivated FBS and 1% penicillin-streptomycin-glutamine (37°C, 5% CO_2_ atmosphere).

The study has received ethical approval by the Institutional Review Board of the University Hospital of Larissa. A written informed consent in accordance with the ethical standards of Helsinki declaration and its later amendments was signed by all the participants prior to sample collection, as well as by parents or relatives of patients for whom consent was not legally available (e.g., children).

### PCR and qPCR

cDNA was prepared from total RNA from mononuclear cells as described (26, 27). Expression of *TACI* and *BAFFR* was determined by quantitative Real-Time RT-PCR, while the expression of *BCMA, APRIL, BAFF*, and deltaBAFF (Δ*BAFF*) transcripts was determined by semi-quantitative conventional RT-PCR using beta-2-microglobulin (B2M) transcripts as reference (Supplementary Methods). TEL-AML1, E2A-PBX1, BCR-ABL (p190 and p210) chromosomal translocations were detected in B-ALL patients by two-step multiplexes RT-PCR as described (28). The methods are fully described in the Supporting Information section.

### Flow Cytometry

Surface expression of BAFFR, BCMA, and TACI was analyzed by flow cytometry with PE-conjugated anti-TACI, anti-BCMA, or anti-BAFFR antibodies (Biolegend, San Diego, CA, USA) and compared to respective isotype-matched Ig controls. Cells were analyzed on Coulter FC-500 flow cytometer (Beckman-Coulter, Brea, CA, USA).

### Chromatin Immunoprecipitations (ChIPs)

Six hundred ninety-seven (697) cells (E2A-PBX1^+^, BAFFR^+^) were crosslinked in culture medium with 1% formaldehyde for 15 min at room temperature. Following sonication, ChIPs were performed according to EZ-ChIP™-Chromatin Immunoprecipitation Kit (Upstate-Millipore, Burlington, Massachusetts, USA) protocol using a mouse anti-E2A-PBX1 antibody (clone G289-1, BD Biosciences, Franklin Lakes, New Jersey, USA) or mouse IgG1 kappa isotype control (BD Biosciences). DNA was amplified by qPCR with primers flanking putative E2A-PBX1 binding sites on *BAFFR* gene (promoter, second intron and 3′UTR) or negative control primers as listed in Table S1. Specific enrichment of E2A-PBX1-binding DNA targets vs. input was calculated as described by Litt et al. (ChIP/Input = 2^InputCt−ChIPCt^) (29).

### Western Blot Analysis

Western blot analysis was performed as described before (30, 31), using anti-NF-κB2 (Millipore), anti-BAFFR (CT; Enzo, Lausen, Switzerland) or anti-actin (Santa Cruz, CA, USA) antibodies. Primary antibodies were visualized with horseradish peroxide-conjugated secondary donkey antibodies using an enhanced chemiluminescence detection system.

### B-Cell Survival Assays

5 × 10^4^ cells were incubated in IMDM-10% FBS in the presence or absence of 60-mer BAFF (0.049–12.5 ng/mL; AdipoGen, San Diego, CA, USA), with/without marimastat (0.5–8 μM, Sigma-Aldrich, St. Louis, Missouri, USA). At the indicated time points (5 and 17 h for B-lymphoblasts and 3 days for normal B-cells, respectively), CD19^+^ 7-AAD^−^cells were analyzed in triplicates by flow cytometry by timed acquisition (10, 30, 31).

### Detection of Cell Death

Six hundred ninety-seven (697) and Jurkat cells were grown in 48-well plates at 10^5^ cells per well in IMDM-10% FBS, in the presence or absence of BAFF 3-mer (5–200 ng/mL; R&D systems, Minneapolis, USA), or 60-mer (5 ng/mL) for 2 days and treated with 3-mer (5–800 ng/mL) or 60-mer BAFF (5 ng/mL) with or without hypotoxic concentrations of aracytine (160 ng/mL; Pfizer, New York, USA), dexamethasone (10–20 ng/mL; Vianex, Athens, Greece), prednisolone (0.8 μg/mL; Takeda, Osaka, Japan), methylprednisolone (1.3 μg/mL; Vianex), hydrocortisone (53 μg/mL;Vianex) for 697 cells and 200 μg/mL dexamethasone for Jurkat cells; according to titration assays, we chose those drug concentrations below the threshold of 50% B-lymphoblasts apoptosis (IC50) to prevent that glucocorticoid-induced cell death would mask potential effects of BAFF. After 72 h, cell death was analyzed using an annexin V cell apoptosis kit (Beckman Coulter); data represent the average of 2–5 experiments.

Based on our findings, 697 cells were also incubated with/without 8 μM of marimastat, 20 ng/mL dexamethasone and 5 ng/mL BAFF for 3 days and apoptosis was assessed as described above.

### Statistical Analysis

Data analysis was performed with SPSS 22.0. Comparisons of gene expression between ALL groups were based on the non-parametric Mann-Whitney *U* and Kruskal-Wallis *H* tests. Correlations at expression level were made according to Spearman's rank correlation coefficient. Statistical significance for *in vitro* cell assays with drugs was tested with Wilcoxon signed-rank test. Graphs were made on Graphpad Prism 6. *P* value (2-sided) <0.05 was considered statistically significant.

## Results

### BAFFR Expression in Acute Lymphoblastic Leukemia Cells and E2A-PBX1 Binding to BAFFR Promoter in Pre-B-lymphoblasts

**Figure 1:**
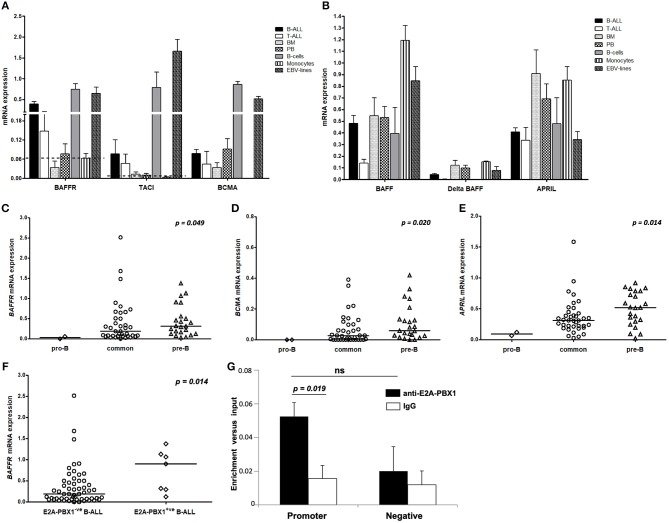
Pattern of mRNA expression of BAFF/APRIL system and E2A-PBX1 binding to *BAFFR* promoter. For **(A–F)** the vertical axis represents the normalized relative gene expression values. **(A)** Receptors and **(B)** ligands mRNA levels in patients with B- (*n* = 63) and T- (*n* = 7) lineage ALL and in mononuclear cells from BM (*n* = 4), PB (*n* = 5), pure mature B-cells (*n* = 3) and pure monocytes (*n* = 3) from healthy donors, and EBV cell lines (*n* = 4). The bars represent mean values and the lines the standard error of mean. The discontinuous lines represent the relative mRNA expression of receptors in monocytes that serve as a negative control group. **(C)** *BAFFR*, **(D)** *BCMA*, and **(E)** *APRIL* mRNA levels are correlated with the maturation stage of B-lymphoblasts. The lines in the dot plots represent the median value for each B-ALL subtype; *p-value* in each plot refers to Kruskal-Wallis *H* test. **(F)** Increased *BAFFR* transcript levels in patients with pre-B-ALL carrying the E2A-PBX1 translocation; *p-value* refers to Mann-Whitney *U* test. **(G)** E2A-PBX1 binding to the promoter of *BAFFR* gene in the pre-B-ALL cell line 697. Cross-linked chromatin from 697 cells was used in ChIPs with anti-E2A-PBX1 antibody. IgG was utilized in parallel with anti-E2A-PBX1 antibody, as control. Two sets of primers were used: One set specific for the amplification of the *BAFFR* gene promoter and one set of negative control primers for amplification of a region lacking PBX motifs. Bars represent mean and standard error values of specific enrichments (fold differences) vs. input obtained in at least three independent experiments. The statistical significance (Student's *t*-tests) of the difference of enrichment for promoter sequences vs. the IgG control (*p* = 0.019) and negative control region (ns, not significant) are indicated.

Analyzing the expression levels of *BAFFR, TACI, BCMA, BAFF*, and *APRIL* relative to *B2M* transcripts, we found that *BAFFR* and *BCMA* transcripts are expressed by B-ALL cells (*n* = 63), although at lower levels compared to primary B-cells or EBV lines, while *TACI* seems to be expressed only at background levels (Figure 1A). Comparing different types of ALL, we detected *BAFFR* expression in 42 out of 63 patients diagnosed as either common ALL or pre-B ALL (Figure 1C); interestingly, the *BAFFR* mRNA expression was correlated with the expression of CD20 (*p* = 0.022, Figure S1A). Moreover, *BAFFR* levels were higher in a proportion of patients carrying the E2A-PBX1 translocation compared to theE2A-PBX1-negative cohort (*p* = 0.014), with all of them suffering from pre-B-ALL (Figure 1F). Similarly to *BAFFR*, we also found aberrant expression of *BCMA* in the more mature forms of B-ALL (Figure 1D), correlating with the expression of cytoplasmic IgM (*p* = 0.027, Figure S1B). Finally, *TACI* transcripts were only observed in patients expressing either *BAFFR* or *BCMA* (*n* = 6); interestingly, when *TACI* was expressed, it was significantly correlated to *BAFFR* mRNA levels (*p* < 0.001, *r* = 0.452, Figure S1C).

Similar to *BAFFR* and *BCMA, APRIL* was expressed by more mature B-ALL cases (Figure 1E) and its mRNA levels were positively correlated with those of *BAFF* (*p* = 0.008, *r* = 0.33). Moreover, we found a significant association of *BAFF* expression with its alternatively spliced isoform Δ*BAFF* (*p* < 0.001, *r* = 0.88, Figure S1D). As Δ*BAFF* interferes with BAFF activity (32), it may play a role as negative regulator for BAFF in B-ALL lymphoblasts. Additionally, we observed that *BCMA* was associated with both Δ*BAFF* (*p* = 0.031, *r* = 0.272) and *APRIL* (*p* = 0.005, *r* = 0.349), while the latter two were also correlated to each other (*p* < 0.001, *r* = 0.434).

Finally, no significant associations were observed between the expression of *BAFF*, Δ*BAFF, APRIL, BAFFR, TACI*, and *BCMA* with clinicolaboratory findings at diagnosis (including leukocyte counts, hemoglobin, and platelet levels), initial response to treatment and survival of our cohort of patients (*p* > 0.05, in all cases).

Since high *BAFFR* mRNA expression was found in about 60% of the E2A-PBX1^+^ pre-B-ALL cases, to functionally investigate the mechanisms underlying this phenomenon, binding of E2A-PBX1 to regulatory sequences in the *BAFFR* locus was analyzed. ChIPs in pre-B-ALL cells (697) that carry the E2A-PBX1 fusion and express only BAFFR, mimicking patients' phenotype, evaluated whether E2A-PBX1 binds *in vivo* to sequences of the *BAFFR* gene that include PBX consensus motifs (promoter, intronic and 3′UTR). E2A-PBX1 was shown to bind to the promoter of *BAFFR*, with the enrichment being 3.3-fold vs. the IgG control (Figure 1G).

**Figure 2:**
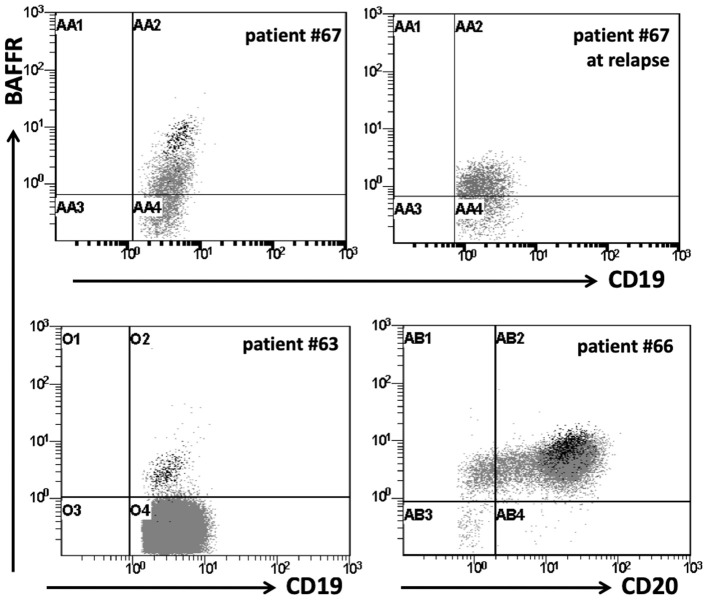
BAFFR expression in patients with B-ALL. Flow cytometry data for surface expression of BAFFR on representative B-ALL patients. BAFFR is expressed in a proportion of patients with B-ALL, but to a lesser degree compared to normal residual mature B-cells. Dot plots show BAFFR expression on total CD19^+^ cells in patients with B-ALL. Black dots represent the residual normal mature B-cells on patients with B-ALL; patients #63 & 66: common B-ALL, patient #67: pre-B-ALL at diagnosis and relapse.

Analyzing the cell surface expression of BAFFR, TACI, and BCMA on B-ALL cells from 12 patients (data for BCMA expression were available for 7/12 patients), we found that B-ALL cells of 8/12 patients expressed BAFFR, although at lower levels than mature B cells from the same patients (Figure 2). Different from B-lymphoblasts, BAFFR is normally not expressed by normal pro-B and pre-B cells (Figure S2). As predicted by the qPCR analysis (Figure 1), neither TACI nor BCMA were expressed by B-ALL cells. Interestingly, induction/consolidation therapy (including GMALL and Hyper-CVAD treatment) did not change BAFFR expression (Figure 2).

### BAFF Activates the Alternative NF-κB Pathway in B-ALL Cells

**Figure 3:**
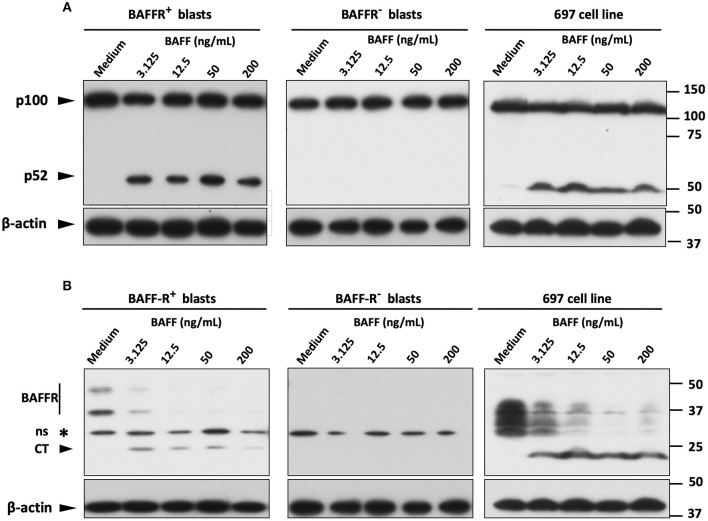
BAFF activates the alternative pathway of NF-κB on BAFFR expressing blasts. **(A)** Western blot analysis of NF-κB2 processing in mononuclear cells from B-ALL patients and in 697 cells, incubated overnight with increasing concentrations of 60-mer BAFF. **(B)** Western blot analysis with the antibody that recognizes also the C-terminal fragment of BAFFR (~22 kDa) showed BAFF-mediated degradation of BAFFR in B-lymphoblasts expressing the receptor. Six hundred ninety-seven cells display an abnormal migration pattern of BAFFR protein (of lower molecular weight) compared to primary B-lymphoblasts (intact BAFFR: ~50 & 36 kDa); ns, non-specific.

Since the NF-κB2 pathway is typically activated by BAFFR (33), we tested the processing of NF-κB2 by stimulating B-lymphoblasts from two BAFFR^+^ pre-B ALL patients and from one BAFFR^−^ common B-ALL patient with BAFF overnight in a dose-dependent manner. The immunoblotting analysis revealed processing of p100 into p52 in BAFFR^+^ pre-B-ALL cells and in the 697 cell line, but not in the BAFFR^−^ common B-ALL sample (Figure 3A), demonstrating that BAFFR expressed in pre-B ALL is functional and initiates NF-κB2 signaling similar to mature B-cells.

Since BAFF binding to BAFFR can induce proteolytic processing of BAFFR by ADAM10 and ADAM17 (30), we tested if BAFFR would also be processed in pre-B ALL cells in response to BAFF binding. Similar to normal B-cells and Burkitt's lymphoma lines (30), a sharp decline of BAFFR was observed in BAFFR^+^ pre-B-ALL and in 697 cells but not in BAFFR^−^ common B-ALL cells (Figure 3B). The decrease of the intact BAFFR protein was accompanied by the appearance of a 22 kDa fragment corresponding to the C-terminal part of BAFFR remaining in the cells after the extracellular part has been shed (30). Thus, BAFF binding to BAFFR regulates the surface expression levels of BAFFR in BAFFR^+^ pre-B ALL cells. However, overnight treatment of 697 cells with the pan-metalloprotease inhibitor marimastat, failed to inhibit BAFFR processing induced by BAFF binding to the receptor (Figure S4A).

### BAFF Promotes Cell Death in B-Lymphoblasts

**Figure 4:**
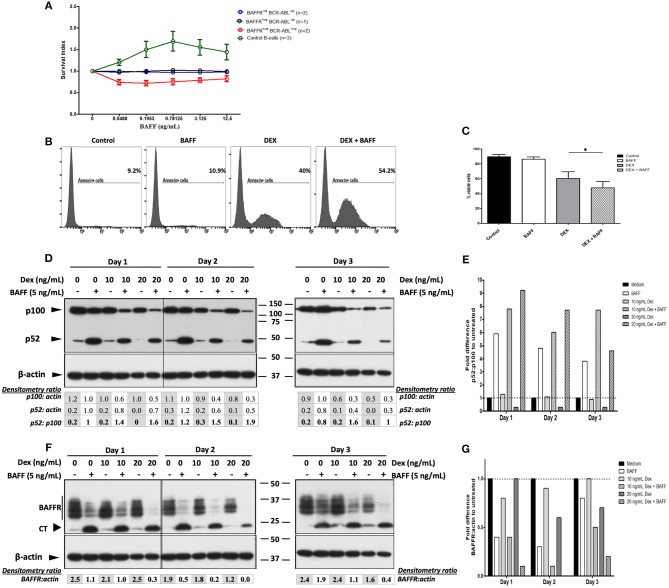
BAFF enhances dexamethasone-induced apoptosis of 697 cells and cell death of BAFFR expressing BCR-ABL^+^ pre-B-lymphoblasts. **(A)** Malignant B-lymphoblasts were incubated with 60-mer BAFF (0.0488–12.5 ng/mL) for 5 and 17 h; the effect of BAFF-treatment in pure normal B-cells that served as a positive control, was evaluated after 3 days. Survival index represents the ratio of 7-AAD^−^CD19^+^ cells at each concentration of the ligand to the untreated 7-AAD^−^CD19^+^ cells. The graph shows the mean and standard error of mean of the survival index at each group (BAFFR^−ve^ BCR-ABL^−ve^: common & pro-B-ALL, BAFFR^+ve^ BCR-ABL^−ve^: common B-ALL, BAFFR^+ve^ BCR-ABL^+ve^: pre-B-ALL, control B-cells: pure normal peripheral B-cells). **(B)** Flow cytometry analysis for Annexin V in 697 cells treated with/without BAFF and with/without dexamethasone at day 5. **(C)** Percentage of viable cell counts (Annexin V^−^ 7-AAD^−^ cells) of 697 cells incubated with/without 3-mer or 60-mer BAFF and with/without 20 ng/mL dexamethasone (DEX) for 5 days (DEX vs. DEX+BAFF *p* = 0.004); *p-value* refers to Wilcoxon signed-rank test between cells treated only with dexamethasone and those treated with dexamethasone and BAFF. **(D)** Western blot analysis of NF-κB2 processing in 697 cells treated with 5 ng/mL 60-mer BAFF and 10–20 ng/mL dexamethasone for 3 sequential days; NA: not applicable. **(E)** Normalization (fold difference) of p52/p100 degradation for each culture condition to that of the untreated cells per each day. **(F)** Western blot analysis of BAFFR (CT) in 697 cells treated with 5 ng/mL 60-mer BAFF and 10–20 ng/mL dexamethasone for 3 sequential days. **(G)** Normalization (fold difference) of intact BAFFR/actin for each culture condition to that of untreated cells per each day.

Since BAFF promotes the survival of transitional and mature B-cells (34), we investigated whether BAFFR expressed by B-lymphoblasts would support their survival *in vitro*. To this end, we performed survival assays with BAFFR^+^ and BAFFR^−^ B-ALL cells isolated from patients and compared their survival, at 5 and 17 h in the presence of increasing amounts of BAFF, using normal B-cells as an internal control. Surprisingly, BAFF did not support the survival of BAFFR^+^ B-lymphoblasts, but it seemed to increase in a dose-independent manner the cell death of BAFFR^+^ pre-B-ALL cells from adult patients carrying the BCR-ABL rearrangement (Figure 4A).

Since BAFF treatment of BAFFR expressing B-lymphoblasts induces processing of the receptor, we wondered whether incubation with marimastat that inhibits BAFFR processing in normal B-cells (30) could modify the effect of BAFF in the survival of primary B-lymphoblasts. However, various concentrations of marimastat did not clearly alter the effect of BAFF in promoting cell death, while there was a clear effect of marimastat on normal B-cells in promoting their survival (Figure S4B).

The unexpected induction of BAFF-dependent cell death on BAFFR^+^ malignant pre-B-lymphoblasts prompted us to investigate whether BAFF treatment would change drug-induced apoptosis of B-ALL cells. Thus, we used the BAFFR^+^ pre-B-ALL cell line 697 as a model system and the BAFFR^−^Jurkat T-ALL cell line as a negative control, due to the lack of expression of BAFF/APRIL receptors. When cells were treated with BAFF and sub-toxic concentrations of drugs used in conventional ALL treatment, like aracytine or the glucocorticoids dexamethasone, prednisolone, hydrocortisone, methylprednisolone, the addition of BAFF significantly enhanced glucocorticoid-dependent apoptosis of 697 cells (Figures 4B,C), but not of Jurkat cells. This effect seemed to be specific for glucocorticoids, since we did not observe any effect of BAFF in aracytine-treated 697 cells (Figure S3). Despite the wide range of BAFF concentrations that were tested, the BAFF-enhanced glucocorticoid-induced apoptosis did not seem to depend on the dose or form (3-mer/60-mer) of the ligand, as high concentrations (200–800 ng/ml) of 3-mer BAFF had similar effects with low concentrations (5 ng/ml) of the 60-mer form (data not shown).

Analysis of NF-κB2 processing (Figures 4D,E) and of BAFFR shedding (Figures 4F,G) revealed that treatment with dexamethasone did neither change the activation of the NF-κB2 pathway nor the proteolytic cleavage of BAFFR, upon treatment with 60-mer BAFF, despite the fact that long-term incubation with dexamethasone itself decreased the levels of p100 and BAFFR. This finding might be due to a repression of the canonical NF-κB pathway that normally supports the generation of p100 and BAFFR (35, 36). The fact that the presence of marimastat did not alter the ability of BAFF to increase cell death induced by dexamethasone (Figure S4C), suggests that treatment with glucocorticoids does not interfere with BAFFR signaling.

## Discussion

Analyzing BAFFR expression by acute lymphoblastic B leukemia cells we found that B-lymphoblasts from patients with common and pre-B-ALL can transcribe the BAFFR-encoding *TNFRSF13C* gene, resulting in substantial surface expression of the receptor. Similar results have been reported by others (16–19), but our data shed new light on these findings since we demonstrated that functional signaling by BAFFR in pre-B ALL cells can increase cell death.

In general, BAFFR expression by B-lymphoblasts was found to correlate with CD20 expression. This expression pattern differs from normal pre-B-cells, in which CD20 expression starts together with or after VDJ rearrangement, whereas low levels of BAFFR are detected first on the surface of immature cells (7). Moreover, BAFFR was found to be expressed at its highest by pre-B ALL cells expressing the chimeric E2A-PBX1 transcription factor. Herein, E2A-PBX1 was shown to bind to *BAFFR* promoter, providing a potential functional implication in the regulation of *BAFFR* expression in pre-B-ALL E2A-PBX1^+^ cells, probably as part of a transcriptional complex, and a framework for further studies.

In mature B-cells, BAFF binding to BAFFR activates NF-κB2 (33). We found that this is also the case in BAFFR^+^ pre-B ALL cells. BAFF-dependent processing of NF-κB2 has been reported for pre-B-ALL cell lines (18) and for primary B-ALL cells (19). Here we show that BAFF binding to BAFFR expressed by pre-B ALL cells activates proteolytic processing of BAFFR and, in contrast to a previous report (19), induces apoptosis. The discrepancy with the study of Parameswaran et al. might be explained by the experimental settings, since Parameswaran et al. analyzed BAFF-dependent responses by growing B-ALL cells together with murine OP9 stroma cells (19), whereas we cultivated purified B-lymphoblasts in the absence of any feeder cells. In the case of normal B-cell precursors developing *in vitro* from CD34^+^ hematopoietic stem cells, the presence of feeder cells may interfere with their normal developmental progress (37). In analogy, the presence of OP9 cells might have provided pro-survival factors which were absent in our pure B-ALL cell culture system.

The unexpected pro-apoptotic function of BAFFR in pre-B ALL cells might be explained by the fact that BAFFR-induced signaling cascades overlap with and enhance BCR-dependent signaling (34, 38–41). Taking into consideration the fact that BCR-ABL overexpression mimics BCR signaling and since enhancement of the signaling cascade downstream of BCR-ABL leads to cell death (12, 42, 43), the activation of BAFFR-dependent signals in pre-B ALL cells might add-up to the tyrosine kinase activity of BCR-ABL (e.g., interfering with other major signaling pathways such as that of PI3K) and provide a rationale for BAFF-induced cell death of these cells. This interpretation is also in line with the observations made by Parameswaran et al. who reported that addition of BAFF reduced the apoptosis rate caused by a BCR-ABL inhibitor (19, 20). BAFF also enhanced glucocorticoid-induced cell death of the 697 pre-B-ALL line without changing the activation of NF-κB2 or BAFFR processing. An increase in the dexamethasone-induced apoptosis of the BAFFR^+^ NALM-6 cell line by BAFF has also been reported in the study of Onda et al. (18), although lacking mechanistic explanation. Our findings together with the fact that marimastat failed to modify the effect of BAFF in dexamethasone-treated cells, suggests that BAFFR and glucocorticoids initiate different pathways, which finally overlap and induce apoptosis of pre-B-cells.

In summary, our data show that the BAFF/APRIL system is functional in B-ALL in a disease-subtype specific manner. Moreover, the participation of E2A-PBX1 in the transcriptional regulation of *BAFFR* sheds more light to the understanding of the deregulated expression of the most predominantly expressed receptor of the BAFF/APRIL system in B-ALL. Since BAFFR activation seems to enhance cell death in BCR-ABL^+^ pre-B ALL cells, monitoring BAFFR expression might provide novel means to classify and finally treat B-ALL.

## Ethics Statement

All experimental procedures performed were approved by the regional ethics committee. A written informed consent in accordance with the ethical standards of Helsinki declaration and its later amendments, was assigned by all the participants prior to sample collection, as well as parents or relatives of patients for whom consent was not legally applicable (e.g., children).

## Author Contributions

ES, HE, and MS provided the study concept and design. ES, EK, CS, E-NK, NA, AG, HE, and MS performed acquisition, analysis, and interpretation of data. MP, NG, GV, MA, MM, and SP provided samples and insight. ES, HE, and MS made drafting of the manuscript. EK, CS, GV, MM, SP, AG, HE, and MS provided critical revision of the manuscript for important intellectual content. ES and MS performed statistical analysis. MS supervised the study.

### Conflict of Interest Statement

The authors declare that the research was conducted in the absence of any commercial or financial relationships that could be construed as a potential conflict of interest.
