# Supplementary material for: BAFF/APRIL System Is Functional in B-Cell Acute Lymphoblastic Leukemia in a Disease Subtype Manner
Source: Front Oncol. 2019 Jul 18;9:594. doi: 10.3389/fonc.2019.00594 (PMC6657364; doi:10.3389/fonc.2019.00594)
Supplement: Supplementary file 1 [file Data_Sheet_1.pdf]

*Supplementary Material*

# BAFF/APRIL System is Functional in B-Cell Acute Lymphoblastic Leukemia in a Disease Subtype Manner


**Eirini Sevdali[1], Eleni Katsantoni[2], Cristian R. Smulski[3], Maria Moschovi[4], Maria Palassopoulou[5], Eleni-Nefeli Kolokotsa[1], Nikoletta Argentou[1], Nikolaos Giannakoulas[5], Maria Adamaki[4], Georgios Vassilopoulos[5], Sophia Polychronopoulou[6], Anastasios E. Germenis[1], Hermann Eibel[3], and Matthaios Speletas[1*]**

[1]Department of Immunology & Histocompatibility, Faculty of Medicine, School of Health Sciences, University of Thessaly, Larissa, Greece

[2]Basic Research Center, Biomedical Research Foundation of the Academy of Athens, Athens, Greece

[3]Center for Chronic Immunodeficiency, University Medical Center Freiburg, Freiburg im Breisgau, Germany

[4]Hematology/Oncology Unit, First Department of Pediatrics, National and Kapodistrian University of Athens, Medical School, "Aghia Sophia" Children's Hospital, Athens, Greece

[5]Department of Hematology, Faculty of Medicine, School of Health Sciences, University of Thessaly, Larissa, Greece

[6]Department of Pediatric Hematology/Oncology, "Aghia Sophia" Children's Hospital, Athens, Greece

*** Correspondence**:

Matthaios Speletas, Department of Immunology & Histocompatibility, Faculty of Medicine, University of Thessaly, Biopolis, Larissa 41500, Greece; e-mail: maspel@med.uth.gr; speletas@gmail.com

## Supplementary Methods

*mRNA analysis*

To detect the transcript levels of *BAFFR* and *TACI*, cDNA was amplified by specific primers in duplicate quantitative Real-time RT-PCR reactions using Platinum-SYBR-Green qPCR Supermix (Invitrogen, Waltham, Massachusetts, USA) in the Rotor-Gene 6000 cycler [Corbett Life Science, Asia-Pacific (APAC), Australasia], and all measurements were averaged, as described elsewhere (1). The *B2M* gene was used as a reference gene, correcting for variations in mRNA recovery and reverse transcription yield. Relative quantification and calculation of the range of confidence were performed using the comparative $\Delta\Delta^{CT}$ method, as described by Livak (2) and the relative expression of each gene is presented as a multiple of the respective gene expression in isolated B-cells of a healthy individual.

*BCMA*, *APRIL*, *BAFF* and *ΔBAFF* mRNA levels were detected by amplifying cDNA products in the presence of 1μM of specific primer in a semi-quantitative conventional RT-PCR using MyTaq DNA polymerase (Bioline, London, UK) in an Applied Biosystems Verity Thermal Cycler (Thermo Fisher Scientific, Waltham, Massachusetts, USA). PCR products were separated on a 2% agarose gel containing ethidium bromide and visualised by the Uvitec gel documentation and analysis system (UVItec Limited, Cambridge, UK) with the aid of the GelCapture acquisition software (DNR Bio-Imaging systems, Neve Yamin, Israel). The quantification of the PCR fragments was performed by the UVIdoc software (UVItec Limited). The expression levels of *BCMA*, *APRIL*, *BAFF* and *ΔBAFF* were calculated as the intensity quotient for each gene to *B2M*.

The primers for the amplification of *TACI*, *BAFF* and *ΔBAFF* were described previously (1, 3), while the primers for the amplification of *BAFFR*, *BCMA*, and *APRIL* were designed with the aid of the Oligo 6.0 software (NBI, Plymouth, MN, USA). The semi-quantitative conventional RT-PCR conditions for each gene were set up so that none of the analyzed RNAs reached a plateau, meaning that they were at the middle of the exponential phase of amplification.

Concerning the detection of TEL-AML1, E2A-PBX1, BCR-ABL (p190 ALL- and p210 CML-type) translocations in patients with ALL, we performed a multiplex two-step RT-PCR as described by Arrifin *et al.* (4). The cell lines K562 (positive for BCR-ABL/p210 rearrangement) and 697 (positive for E2A-PBX1 rearrangement), as well as patients' samples with previously established rearrangements for BCR-ABL/p190 and TEL-AML1 were used as positive controls in our experiments. For the confirmation of results, all PCR products with suspected rearrangements were purified by QIAquick gel extraction kit (Qiagen, Valencia, CA, USA) and directly sequenced using an ABI Prism 310 Genetic Analyzer (Applied Biosystems, Foster City, CA, USA) and a Big Dye Terminator DNA sequencing kit (Applied Biosystems).

*Cell isolation*

For B-cell survival assays, B-ALL lymphoblasts from patients and normal peripheral blood B-cells were enriched from mononuclear cells by magnetically depleting all non-B-cells. In particular, cells were incubated with FITC-labelled antibodies against CD2, CD3, CD14, CD16, CD36, CD43 (Beckman Coulter, Brea,

CA, USA) with the exception of patients with B-ALL, where the anti-CD43 antibody was not included in the staining, as malignant B-lymphoblasts express the molecule. Afterwards, all non-B-cell subsets were positively isolated with the aid of the EasySep™ Human FITC Positive Selection Kit (Stemcell Technologies, Vancouver, USA). Purity of the isolated B-cells (malignant or not) was assessed by flow cytometry with a PE-labelled anti-CD19 antibody (Beckman Coulter).

For the measurement of mRNA levels of BAFF/APRIL ligands and receptors, B-cells and monocytes were also isolated from PBMCs of healthy donors. B-cells were isolated after labelling with a PE-conjugated anti-CD19 antibody (Beckman Coulter), followed by positive selection with EasySep™ Human PE Positive Selection Kit (Stemcell Technologies). For monocyte isolation, cells were negatively selected using the FITC-conjugated antibodies CD2, CD4, CD8, CD19, CD43 (Beckman Coulter) and the EasySep™ Human FITC Positive Selection Kit that was mentioned above.

## Supplementary Tables

Supplementary Table 1. Primers used in this study.

| Gene | Forward primer(5' →3') | Reverse primer(5' →3') |
|---|---|---|
| **Quantitative Real Time RT-PCR** | | |
| *BAFFR* | GAGACAAGGACGCCCCAGA | GGTCACCAGTTCAGTGGAGC |
| *TACI* | TCTGCCTGTGTGCCGTCCTC | CGGCTTCCATCGCGTGAT |
| *B2M* | Commercially obtained by SAbiosciences (cat no. PPH01094E) | |
| **Semi-quantitative conventional RT-PCR** | | |
| *BCMA* | TTGGGACTGAGCTTAATAAT | CGGAAGAATAATTTCATCAC |
| *BAFF & ΔBAFF* | GAGTCTCCCGGAGCAGAGTT | GCAGGAATTATTGGGTAGTGT |
| *APRIL* | GAGTCTCCCGGAGCAGAGTT | CTTGGAGGTGGCGTTAATGG |
| *B2M* | CTATCCAGCGTACTCCAAAG | GAAAGACCAGTCCTTGCTGA |
| **Real Time PCR for ChIP** | | |
| *BAFFR promoter* | GGCGCTATCTCGGCTCA | GCGCCTACAATCTCAGCTAC |
| *BAFFR* $2^{nd}$ *intron* | GGGCAGGGAGTTGGGG | TTGGGAGTCTAACGGAGCTT |
| *BAFFR 3'UTR* | AAATGGCATCTTAGGTTCTG | GCTTGCTTTGGGATTTAGT |
| *Negative control region* | CCCATGAAGACCCAGGCAAG | GGCGAGTCCAGAGCAGTGTG |

**Supplementary Table 2. BAFF promotes cell death of B-lymphoblasts from adult patients with BCR-ABL$^{+}$ pre-B-ALL.** Isolated blasts from 5 patients with B-ALL were cultured for 5 and 17 hours with 60-mer BAFF (0.049-12.5 ng/mL). Normal peripheral CD19$^{+}$ cells were evaluated after 3 days, serving as positive controls; each condition was tested in triplicates. The number of CD19$^{+}$ 7AAA-D$^{-}$ cells was determined by flow cytometry (mean ± standard deviation); patient 64: BAFFR$^{-}$ common B-ALL, patient 71: BAFFR$^{+}$ common B-ALL, patients 65 & 69: BAFFR$^{+}$ BCR-ABL$^{+}$ pre-B-ALL, patient 72: BAFFR$^{-}$ pro-B-ALL, controls 1-3: CD19$^{+}$ cells isolated from peripheral blood mononuclearcells of 3 healthy donors.

| | | **BAFF (ng/mL)** | | | | |
|---|---|---|---|---|---|---|
| **Patient no.** | **Control** | **0.049** | **0.195** | **0.781** | **3.125** | **12.5** |
| **Patient 64 (5h)** | 11100 ± 315 | 11686 ± 742 | 11343 ± 356 | 11025 ± 57 | 11093 ± 172 | 11135 ± 127 |
| **Patient 64 (17h)** | 10044 ± 51 | 10523 ± 287 | 10495 ± 224 | 10551 ± 88 | 10106 ± 152 | 10116 ± 174 |
| **Patient 71 (5h)** | 10768 ± 564 | 10254 ± 373 | 10627 ± 408 | 11012 ± 523 | 10489 ± 83 | 10458 ± 107 |
| **Patient 71 (17h)** | 7471 ± 129 | 7394 ± 135 | 7564 ± 64 | 7626 ± 353 | 7766 ± 325 | 7569 ± 405 |
| **Patient 65 (5h)** | 2354 ± 24 | 1697 ± 32 | 1791 ± 134 | 1937 ± 104 | 1964 ± 93 | 2014 ± 43 |
| **Patient 65 (17h)** | 478 ± 35 | 361 ± 11 | 352 ± 20 | 420 ± 18 | 391 ± 52 | 448 ± 38 |
| **Patient 69 (5h) – 1st experiment** | 6412 ± 591 | 5692 ± 114 | 5358 ± 220 | 5527 ± 142 | 5575 + 69 | 5522 ± 114 |
| **Patient 69 (17h)– 1stexperiment** | 2295 ± 65 | 1281 ± 32 | 982 ± 90 | 881 ± 124 | 1278 ± 417 | 888 ± 185 |
| **Patient 69 (5h)– 2nd experiment** | 6346 ± 221 | 5766 ± 64 | 5544 ± 230 | 5668 ± 289 | 5901 ± 91 | 6138 ± 117 |
| **Patient 69 (17h)– 2nd experiment** | 2453 ± 40 | 1269 ± 265 | 1221 ± 60 | 1257 ± 40 | 1469 ± 51 | 1685 ± 90 |
| **Patient 72 (5h)– 1stexperiment** | 11939 ± 71 | 12450 ± 346 | 11740 ± 331 | 11855 ±303 | 11779 ± 196 | 11529 ± 91 |
| **Patient 72 (17h)– 1stexperiment** | 7709 ± 508 | 7251 ± 118 | 7147 ± 144 | 6990 ± 102 | 7215 ± 223 | 7078 ± 140 |
| **Patient 72 (5h)– 2nd experiment** | 11474 ± 123 | 11189 ± 229 | 11366 ± 777 | 11129 ± 99 | 11449 ± 153 | 11868 ± 286 |
| **Patient 72 (17h)– 2nd experiment** | 8251 ± 420 | 7563 ± 98 | 7281 ± 150 | 7310 ± 324 | 7566 ± 126 | 7777 ± 120 |
| **Patient 69 (5h) after induction therapy** | 6155 ± 185 | 5817 ± 35 | 5799 ± 256 | 5830 ± 105 | 5823 ± 140 | 6170 ± 232 |
| **Patient 69 (17h) after induction therapy** | 2433 ± 165 | 1616 ± 114 | 1580 ± 190 | 1685 ± 186 | 1875 ± 164 | 2087 ± 137 |
| **Control #1** | 1797 ± 133 | 2407 ± 155 | 3378 ± 98 | 3855 ± 149 | 3442 ± 405 | 3216 ± 191 |
| **Control #2** | 5986 ± 762 | 6628 ± 156 | 7620 ± 112 | 8415 ± 324 | 8013 ± 186 | 7172 ± 149 |
| **Control #3** | 3390 ± 147 | 3900 ± 144 | 4611 ± 70 | 5107 ± 72 | 4750 ± 220 | 4466 ± 107 |

## Supplementary Figures

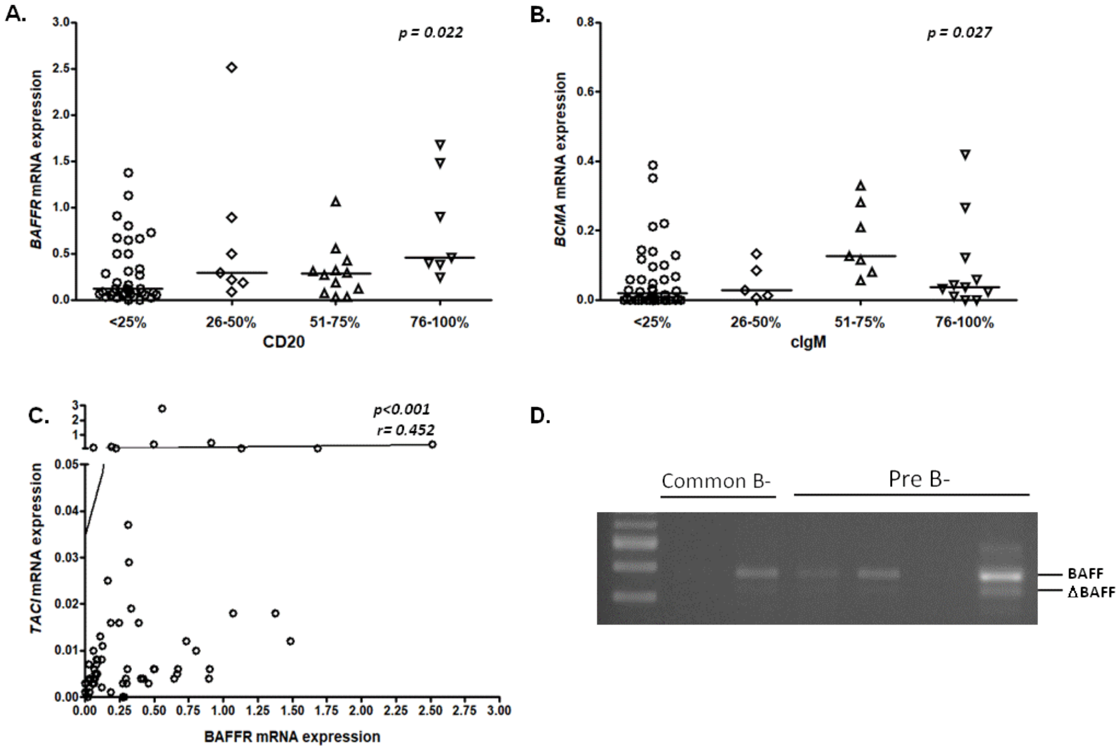


**Supplementary Figure 1. Correlation of receptors expression at mRNA level. (A)** *BAFFR* mRNA levels are correlated with the surface expression (%) of CD20. **(B)** *BCMA* mRNA levels are correlated with the expression (%) of cytoplasmic IgM; *p-value* in each plot refers to Kruskal-Wallis *H* test. **(C)** *TACI* mRNA levels are positively associated with *BAFFR* mRNA levels; p-value refers to Spearman's rank correlation coefficient. **(D)** Representative data of mRNA expression of *BAFF* and *ΔBAFF* in patients with common and pre-B-ALL.

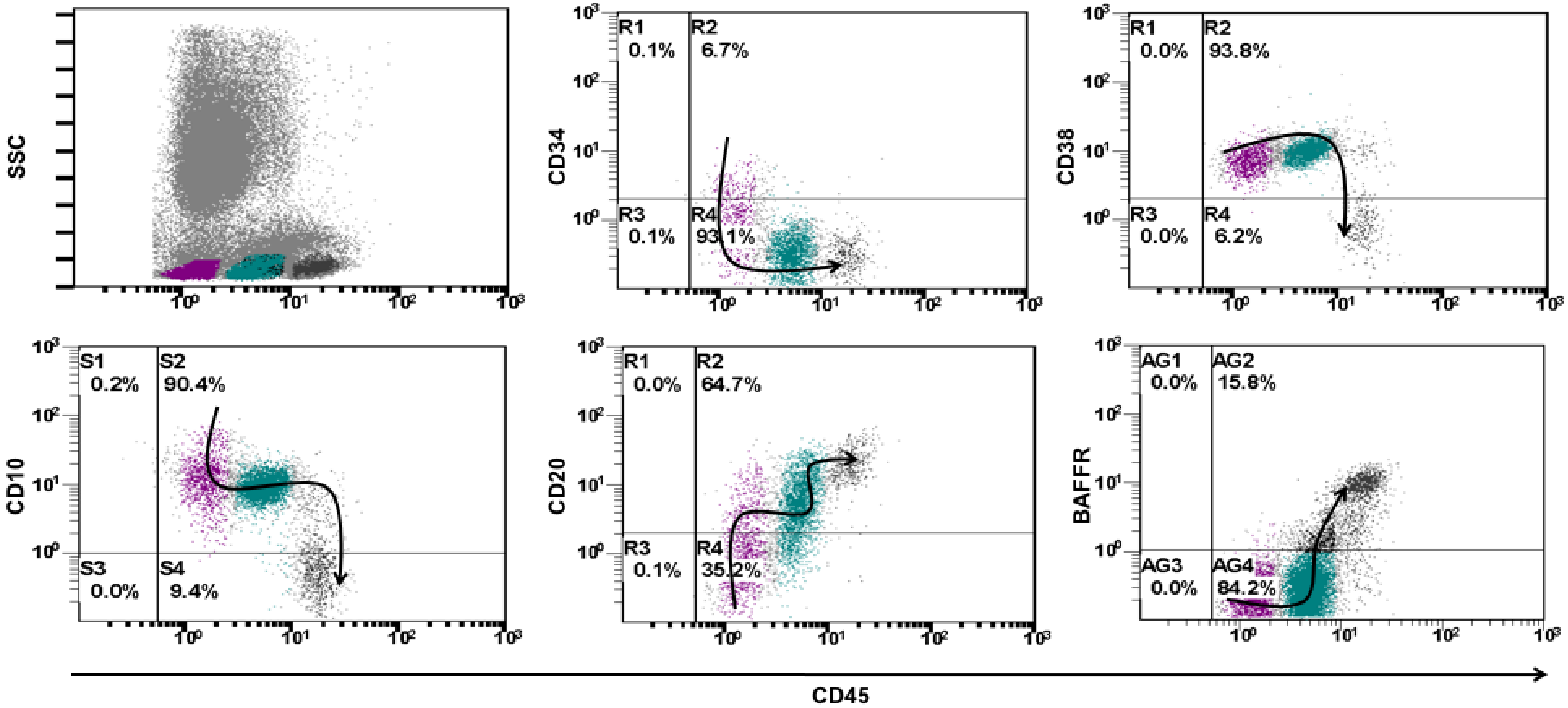


**Supplementary Figure 2. Representative expression pattern of antigens during B-cell maturation.** Dot plots are gated in CD19$^{+}$ cells. The arrows show the direction of maturation pathway. BAFFR is not expressed in normal early stage B-cell progenitors.

Violet cells: CD34+ CD45low, CD38hi, CD10hi CD20lo/- BAFFR- (CLP and pro-B cells)

Green cells: CD34- CD45int, CD38hi, CD10hi, CD20 int, BAFFR- (pre-B and immature B cells)

Black cells: CD34- CD45+/high, CD38low, CD10-, CD20+ BAFFR+ (recirculating mature B cells)

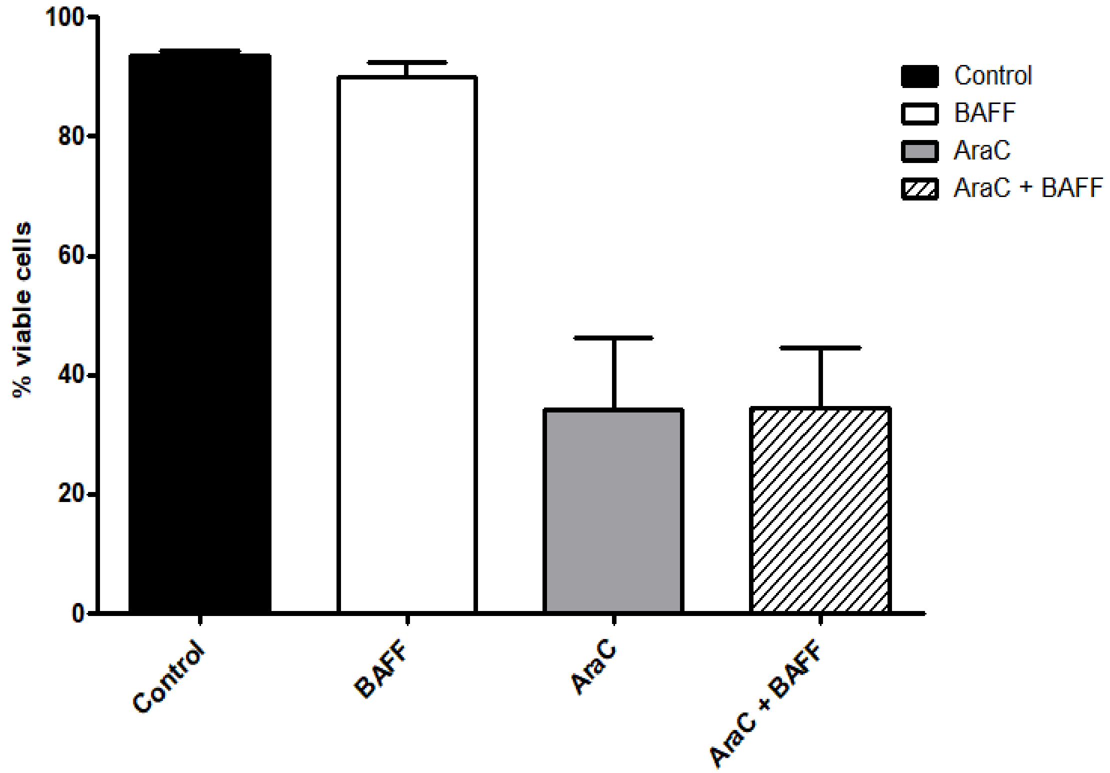


**Supplementary Figure 3. BAFF does not affect the aracytine-mediated apoptosis of 697 cells.** Percentage of viable cell counts (Annexin V$^-$ 7-AAD$^-$ cells) of 697 cells incubated with/without 3-mer BAFF and with/without 160 ng/mL aracytine (AraC) for 5 days.

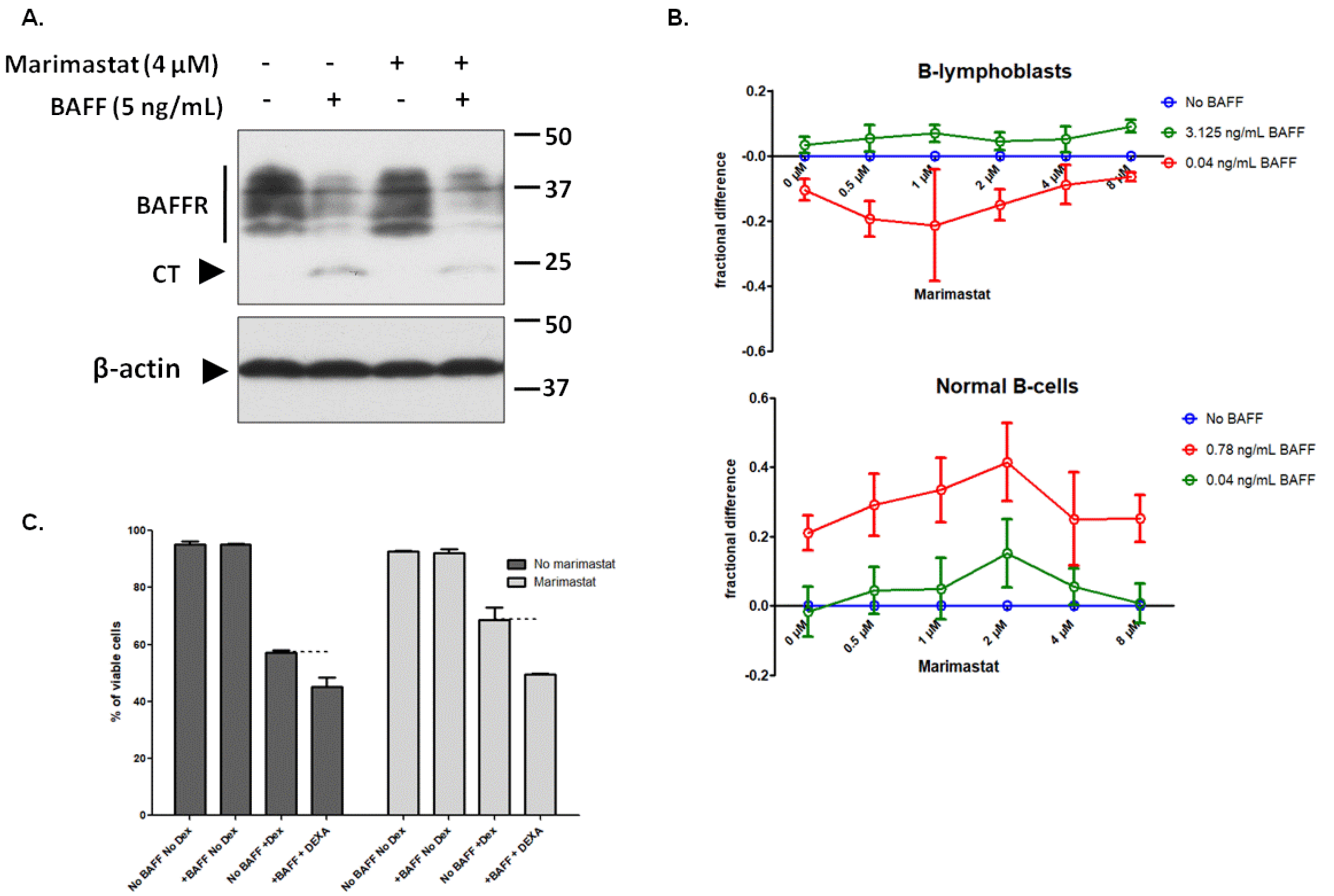


**Supplementary Figure 4. Marimastat does not alter the BAFF-mediated spontaneous or dexamethasone-induced apoptosis of primary B-lymphoblasts and 697 cells, respectively. (A)** 697 cells were incubated overnight with/without 4 µM Marimastat and with/without 5 ng/mL 60-mer BAFF. Cell lysates were blotted using an antibody that detects the C-terminal (CT) fragment of BAFFR. **(B)** Primary B-lymphoblasts of

a BAFFR$^+$ BCR-ABL$^+$ pre-B-ALL patient and pure normal B-cells were treated with increasing concentrations of marimastat and 2 concentrations of 60-mer BAFF (one optimal and one suboptimal according to previous survival assays). At the indicated time points (5h & 17h for B-lymphoblasts, 3 days for normal B-cells), the number of CD19$^+$ 7-AAD$^-$ cells was evaluated. Plots show the fractional difference [calculated as (cell number in the presence of BAFF - cell number w/o BAFF) / cell number w/o BAFF, for each concentration of marimastat respectively] between treatment conditions in B-lymphoblasts (17h) and in pure normal B-cells. **(C)** 697 cells were incubated for 3 days with/without 8 μM marimastat, in the presence or not of 60-mer BAFF (5 ng/mL) and/or 20 ng/mL dexamethasone (dex). Despite the fact that marimastat opposes to dexamethasone action, 697 cells retain their ability to display increased cell death in response to BAFF and dexamethasone treatment, regardless of the presence of the pan-metalloprotease inhibitor. Plots show mean of percentage of viable cell counts (Annexin V$^-$ 7-AAD$^-$ cells) of 697 cells.

## References

1. Mamara A, Germenis AE, Kompoti M, Palassopoulou M, Mandala E, Banti A, et al. TACI expression and signaling in chronic lymphocytic leukemia. J Immunol Res (2015) 2015:478753.

2. Livak KJ, Schmittgen TD. Analysis of relative gene expression data using real-time quantitative PCR and the 2(-Delta Delta C(T)) Method. Methods (2001) 25:402-8.

3. Novak AJ, Slager SL, Fredericksen ZS, Wang AH, Manske MM, Ziesmer S, et al. Genetic variation in B-cell-activating factor is associated with an increased risk of developing B-cell non-Hodgkin lymphoma. Cancer Res (2009) 69:4217-24.

4. Ariffin H, Chen SP, Wong HL, Yeoh A. Validation of a multiplex RT-PCR assay for screening significant oncogene fusion transcripts in children with acute lymphoblastic leukaemia. Singapore Med J (2003) 44:517-20.
